# Excess of Women

**Published:** 1920-02-14

**Authors:** 


### THE PUBLIC WELL-BEING.

### Excess of Women.

DR. MURRAY-LESLIE'S lecture at the Institute of Hygiene—which has been providing material of unusual interest in its recent meetings—has set all the daily press on to the subject of the economic, moral, and social effects of the excess population of women over men. Leading articles and interviews from various points of view have appeared, and, though many analyses of the consequences have been made, there are no easy remedies. Dr. Murray-Leslie drew many shrewd deductions from the fact of women's preponderance, and gave some curious details which led him to two main conclusions—the encouragement of women's emigration and an effort to reduce the male infant mortality, which is greater than female infant mortality.

In all countries there are more boy babies than girl babies born, but the proportion of boys to girls in this country is, for some strange reason, not so large as in other lands. Boy babies, however, are more difficult to bring up—one medical man declares that it is because they are higher up in the scale of creation than girls—and women adults of productive age exceed males by over one million. The war losses of potential fathers have accentuated the difference, and Dr. Murray-Leslie's view is that this large excess of women is responsible for social discontent. This seems probable. There were many who attributed the unseemly antics of the "suffragettes" to the passionate discontent of a certain section of womanhood, and it is altogether reasonable that, if the natural instincts of women towards the joys of motherhood are denied fulfilment through the sheer logic of hard facts and figures, there may accompany this denial a certain bitterness of disappointment and discontent. It is true that the greater economic chances for women nowadays form an outlet for the nervous energies, and many of the finest social workers have been and are unmarried women. But it would be idle to pretend—despite those who insist on being described as "bachelor women"—that, in general, women are indifferent to the fact that so many must perforce forgo one, at least, of woman's highest destinies.

If the number of unmarried women continued to increase there would have to be a still greater adjustment of economic conditions. But there is another feature of this problem to which Dr. Murray-Leslie drew attention—namely, the moral effect of this state of things. The baser sort of women act with an irresponsibility that, Dr. Murray-Leslie declares, is the cause of so many unhappy marriages. Young men also are being spoiled by the inveiglings of butterfly girls, with the result that they are superficially attracted, and many of the best types of womanhood stay outside the marriage circle. The result is that the race is not being recruited from the best stock, and the largest families are those of parents least able, either on grounds of intelligence or economic position, to bring up their children properly.

It is easier to recommend the remedy of women's emigration than to have it carried into effect, and the most hopeful line of progress is to intensify the efforts to reduce infant mortality. As there are more boy babies than girls, the general reduction of mortality will of itself tend towards redressing the balance, and here is to be seen one phase of the vital importance of all infant and child welfare.

One of the most striking proofs of the value of educational work, at baby centres and elsewhere, is supplied by the falling infant death-rate in London, where intensive methods have been applied. Last year it was 85 per 1,000 births, or nearly 10 per 1,000 less than that in the 96 big towns of the country. London is doing more than the other cities to save the boys and equalise the sexes.

